# Long-Term Reproducible Expression in Human Fetal Liver Hematopoietic Stem Cells with a UCOE-Based Lentiviral Vector

**DOI:** 10.1371/journal.pone.0104805

**Published:** 2014-08-12

**Authors:** Niraja Dighe, Maroun Khoury, Citra Mattar, Mark Chong, Mahesh Choolani, Jianzhu Chen, Michael N. Antoniou, Jerry K. Y. Chan

**Affiliations:** 1 Experimental Fetal Medicine Group, Department of Obstetrics and Gynecology, Yong Loo Lin School of Medicine, National University of Singapore, Singapore, Singapore; 2 Interdisciplinary Research Group in Infectious Diseases, Singapore-Massachusetts Institute of Technology Alliance for Research and Technology, Singapore, Singapore; 3 Division of Bioengineering, School of Chemical and Biomedical Engineering, Nanyang Technological University, Singapore, Singapore; 4 Koch Institute for Integrative Cancer Research and Department of Biology, Massachusetts Institute of Technology, Cambridge, Massachusetts, United States of America; 5 Department of Medical and Molecular Genetics, King's College London School of Medicine, Guys Hospital, London, United Kingdom; 6 Department of Reproductive Medicine, KK Women's and Children's Hospital, Singapore, Singapore; 7 Cancer and Stem Cell Program, Duke-NUS Graduate Medical School, Singapore, Singapore; University of Colorado Denver, United States of America

## Abstract

Hematopoietic Stem Cell (HSC) targeted gene transfer is an attractive treatment option for a number of hematopoietic disorders caused by single gene defects. However, extensive methylation of promoter sequences results in silencing of therapeutic gene expression. The choice of an appropriate promoter is therefore crucial for reproducible, stable and long-term transgene expression in clinical gene therapy. Recent studies suggest efficient and stable expression of transgenes from the ubiquitous chromatin opening element (UCOE) derived from the human *HNRPA2B1-CBX3* locus can be achieved in murine HSC. Here, we compared the use of *HNRPA2B1-CBX3* UCOE (A2UCOE)-mediated transgene regulation to two other frequently used promoters namely EF1α and PGK in human fetal liver-derived HSC (hflHSC). Efficient transduction of hflHSC with a lentiviral vector containing an *HNRPA2B1-CBX3 UCOE*-eGFP (A2UCOE-eGFP) cassette was achieved at higher levels than that obtained with umbilical cord blood derived HSC (3.1x; p<0.001). While hflHSC were readily transduced with all three test vectors (A2UCOE-eGFP, PGK-eGFP and EF1α-eGFP), only the A2-UCOE construct demonstrated sustained transgene expression *in vitro* over 24 days (p<0.001). In contrast, within 10 days in culture a rapid decline in transgene expression in both PGK-eGFP and EF1α-eGFP transduced hflHSC was seen. Subsequently, injection of transduced cells into immunodeficient mice (NOD/SCID/*Il2rg* ^-/-^) demonstrated sustained eGFP expression for the A2UCOE-eGFP group up to 10 months post transplantation whereas PGK-eGFP and EF1α-eGFP transduced hflHSC showed a 5.1 and 22.2 fold reduction respectively over the same time period. We conclude that the A2UCOE allows a more efficient and stable expression in hflHSC to be achieved than either the PGK or EF1α promoters and at lower vector copy number per cell.

## Introduction

Hematopoietic stem cells (HSCs) are characterised by their ability to home, engraft and reconstitute the entire hematopoietic system. As such, they represent an ideal vehicle for the life-long delivery of gene products, particularly in the treatment of blood disorders [1]. Many genetic diseases are now being considered as candidates for HSC-based gene therapy, including severe combined immune deficiency (SCID) conditions, lysosomal storage diseases, hemophilias, haemoglobinopathies (β-thalassemia, sickle cell disease) and Wiskott-Aldrich syndrome (WAS). HSC are conventionally obtained from bone marrow aspirates, but is associated with lack of donors and significant donor site morbidity. Nevertheless, successful outcomes from *ex vivo* genetic correction and transplantation of autologous HSC have been reported for SCID-X1 [2]–[4], SCID-ADA [5], X-linked adrenoleukodystrophy (ALD) [6]. Over the past two decades, umbilical cord blood (UCB) has emerged as an attractive and established source for allogeneic and autologous transplantation [7]. Indeed, UCB-HSCs have been studied as potential vehicles for gene delivery in recent years [8], [9]. A major limitation, however, is the low transduction efficiency inherent to HSC. Thus, several research groups have developed novel protocols to improve gene transfer efficiency, with varying results [10]. Our group has previously demonstrated that fetal stem cells are more amenable to lentiviral vector transduction than their adult counterparts [11]. Extending on this theme, we describe here the isolation of fetal-liver HSC from different gestational ages, and evaluate the use of such HSC for gene delivery applications.

Integrating gammaretroviral (RV) and lentiviral (LV) vectors have been utilized in long-term expression of therapeutic transgenes [12]-[15]. However, silencing of transgenes either due to DNA methylation or histone modifications is a cause of concern [16], [17]. Elements with an insulator or boundary function have been used in both RV and LV in an effort to overcome the effects of promoter-dependent silencing of transgene expression, which serve in some cases as barriers to protect against the incursion of adjacent inactive condensed chromatin. For instance, the chicken β-globin locus control region element HS4 (cHS4) has been used in flanking transgenes. But often, these have resulted in limited efficiency thereby compromising their utility for gene delivery applications [18], [19].

Studies have shown the ability of the ubiquitous chromatin opening element (UCOE) consisting of the methylation-free CpG island encompassing the dual divergently transcribed promoters of the human *HNRPA2B1-CBX3* housekeeping genes (A2UCOE) to be able to drive stable and long-term transgene expression [16]. Stable expression from the A2UCOE can be achieved from either its innate HNRPA2B1 promoter [20] or by shielding linked tissue-specific or constitutive [21], [22], [23] heterologous promoters from epigenetic modifications and chromosomal position effects and thus the A2UCOE shows its potential use as an excellent regulatory element in gene transfer studies. A2UCOE driven expression has been successfully employed to stabilize transgene expression in murine hematopoietic stem and peripheral blood cells [20], [21] and in several murine and human iPS and ES cell lines where stable expression was maintained in their progeny including cardiac and hematopoietic differentiated cells [22], [23].

In this study, we have investigated if the A2UCOE can be used to provide stable expression in human fetal liver-derived HSC (hflHSC). Furthermore, we compared A2UCOE efficacy with two other widely used promoters, elongation factor 1α (EF1α) and phosphoglycerate kinase 1 promoter (PGK), using both *in vitro* and *in vivo* HSC repopulating assays in mice. Our results show that the A2UCOE can provide stable, long-term expression whereas the EF1α and PGK promoters are prone to silencing in both assay systems.

## Materials and Methods

### Plasmids and production of lentiviral vector stocks

The PGK-eGFP and EF1α-eGFP plasmids were obtained from Addgene, and the A2UCOE-eGFP vector was as previously described [20]. Lentiviral vector (LV) stocks were generated by triple plasmid co-transfection of HEK293T cells, with a Calcium Phosphate Transfection Kit (Invitrogen, USA) as previously described [11]. The envelope plasmid pMD.G and packaging plasmid pCMV8.91 have been described previously [24]. A total of 30 µg of plasmid DNA was used for the transfection of a single 75 cm^2^ flask: 5.25 µg of envelope plasmid, 9.75 µg of packaging plasmid and 15 µg of transfer vector plasmids (A2UCOE-eGFP, PGK-eGFP or EF1α-eGFP). The medium was replaced with DMEM supplemented with 10% heat inactivated Fetal Bovine Serum (FBS) 24 hrs after transfection. At 48 and 72 hrs after transfection the medium was harvested and passed through a 0.22 µm nitrocellulose filter. Vector particles were concentrated 300 fold by ultracentrifugation at 50,000 g (26,000 rpm with a SW28 rotor) for 140 mins at 4°C and resuspended in 1% BSA in PBS. Viral titers were established by transducing HEK293T cells with serial dilutions of virus stocks and monitoring expression after 3 days by flow cytometry.

#### Ethics Statement

Collection of human tissues from second trimester fetuses and umbilical cord blood from normal full term deliveries was approved by the Domain Specific Review Board of the National University Hospital (Singapore). In all cases, patients gave separate written consent for the use of the collected tissue. All animal protocols were approved and followed in accordance to guidelines by Office of Safety, Health and Environment (OSHE) and Institutional Animal Care and Use Committee (IACUC) at the National University of Singapore.

### Human cells and CD34+ cell purification

Umbilical cord blood was collected from term deliveries (n = 9). Fetal liver samples were obtained from 16 fetuses (median gestational age 17 weeks; range 13 to 22 weeks). Single cell suspensions were prepared by mincing the liver through a 100 µm cell strainer, following incubation with collagenase IV (2 mg/ml, Gibco, USA) in DMEM. Cells collected were centrifuged at 375 g for 10 minutes; the pellet was subjected to red blood cell lysis (ACK lysing buffer, Invitrogen, USA) and washed in DMEM. MNCs were then counted and CD34+ cells were isolated from second trimester human livers and umbilical cord blood with RosetteSep system using the CD34 positive selection kit (Stem Cell Technologies, Singapore).

### Transduction of hematopoietic stem cells

LV containing eGFP under control of the A2UOCE, EF1α and PGK were used to transduce CD34^+^ cells harvested from human fetal liver samples at a different multiplicity of infection (MOI) (MOI is the number of viral particles added per cell seeded for transduction). HSC were suspended at a density of 1×10^6^ cells/ml in transduction cocktail (serum free IMDM (Gibco, USA) containing BIT serum substitute (Stem Cell Technologies, Singapore), 20 ng of recombinant human interleukin-6 (rhIL-6) per ml (R&D systems, USA), 20 ng of thrombopoietin/ml (Peprotech, USA), 100 ng of stem cell factor (rhSCF) per ml (R&D systems, USA) and 100 ng of FLT3-L/ml) (Peprotech, USA) for 24 hrs. Cells were then infected at multiplicities of infection (MOI) of 1, 5, 10, 15 & 20 with A2-UCOE-eGFP, PGK-eGFP and EF1α-eGFP in presence of 4 µg/ml polybrene. Cells were resuspended and cultured in a serum free defined expansion medium as described previously [25], 24 hrs after transduction. Transduced cells at MOI 10, 15 and 30 for A2-UCOE, PGK and EF1α lentiviruses were collected on day 3, 10, 17 and 24 of continuous liquid culture and eGFP expression was analyzed by FACS. All transduction events for each biological sample and infection were performed in triplicates.

### Hematopoietic colony forming cell assays

Vector transduced CD34+ cells were plated in 1 ml of methylcellulose medium (HSC-CFU media) supplemented with FBS, Bovine Serum Albumin (BSA), and different growth factors (e.g. GM-CSF, G-CSF, SCF, IL-3, IL-6, and Epo (Miltenyi Biotec, USA) and was performed in duplicate. Hematopoietic colony forming units (CFU-E, BFU-E, CFU-G and CFU-M) were scored after 14 days of culture. eGFP (fluorescent) colonies were identified by fluorescence microscopy.

### Hematopoietic reconstitution in a murine model

NSG (NOD/SCID/*Il2rg* ^-/-^) mice were obtained from Jackson Laboratory and maintained under specific pathogen-free conditions in the animal facility at the National University of Singapore. Pups were sublethally irradiated within 48 hrs of birth, and injected intracardially with 2×10^5^ lentiviral vector-transduced hflHSCs per pup as previously reported [25]. Peripheral blood was analysed from 3 months to 10 months after transplantation by flow cytometry for eGFP expression and human-specific leukocyte markers using antibodies specific for CD3, CD19 and CD33 (Biolegend, Singapore). Bone marrow was analysed for eGFP expression at 10 months post transplantation.

### Determination of vector copy number by quantitative PCR

Genomic DNA was extracted from cells at Day 17 of culture using the DNeasy kit (Qiagen, USA). 15 ng of genomic DNA was subjected to quantitative PCR using 1xSYBrGreen Master Mix and 100 nM of each eGFP forward primer, 5′- GGCATCGACTTCAAGGAG and reverse primer, 5′- ATAGACGTTGTGGCTGTT that amplified a 74 bp region of the eGFP sequence. A 294 bp region of the human β-actin gene was amplified using forward primer 5′- TCACCCACACTGTGCCCATCTACGA-3′ and reverse primer 5′- CAGCGGAACCGCTCATTGCCAATGG-3′ as a loading control.

### Chimerism

Levels of engraftment were ascertained in 19 animals from the A2UCOE (n = 6), PGK (n = 8) and EF1α (n = 5) groups. Chimerism was calculated as Chimerism  =  %CD45^+^ human cell/(%CD45^+^ human cell + %CD45^+^ mouse cell).

### Statistical Analysis

Parametric data are shown as mean ± standard deviation, and were analyzed using one-way or two-way analysis of variance followed by the Bonferroni Test. Nonparametric data were shown as median and range, and analyzed using the Mann–Whitney Test. A p value of <0 .05 was considered significant.

## Results

### High level presence and LV transduction efficiency of human fetal HSC

**Figure 1:**
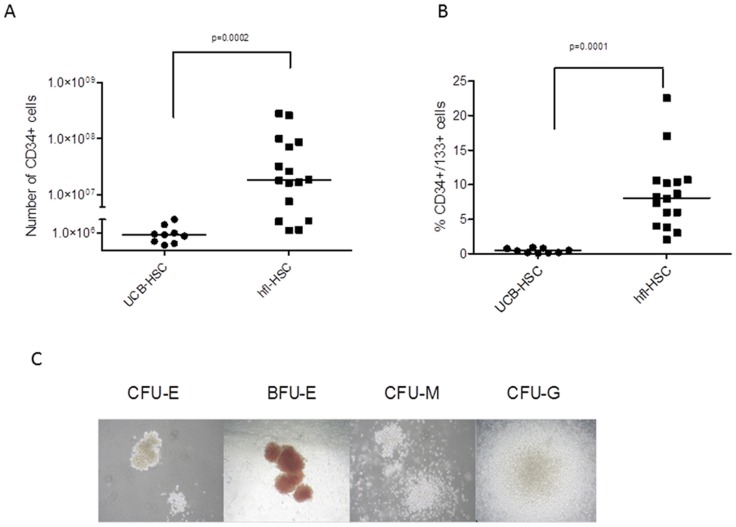
Characterisation of Human Fetal Liver HSC. A & B) Human fetal liver haematopoietic stem cell (hflHSC) populations contain a higher proportion of CD34+ cells than that found in umbilical cord blood (UBC) (1.8×10^7^ versus 8×10^5^; p = 0.0002 and 8.1% versus 0.4%; p = 0.0001). (C) Representative images of multilineage differentiation of hflHSC in methylcellulose culture assays.

The number of CD34+ cells present in fetal livers was observed at a median of 1.8×10^7^ (n = 16; range 1.4×10^6^ to 2.8×10^8^) between 13 to 22 weeks. This is in comparison to a median of 8×10^5^ (n = 9; range 8×10^5^ to 6×10^6^) (p = 0.0002) CD34+ cells typically recovered from a standard UCB collection of around 80-100 ml (Figure 1A). HSCs co-expressing CD34 and CD133, which are markers linked to HSC repopulating activity [26], were observed at a median of 8.1% in fetal liver-derived cells (n = 16, range 2.0 – 22.5%; gestation 13–22 weeks) compared to a median of 0.4% in UCB (n = 9; range 0.1–0.9%) (p = 0.0001) (Figure 1B). Furthermore, human fetal HSCs showed ability of multilineage differentiation into erythroid, granulocytes and myeloid lineages in methylcellulose culture assays (Figure 1C).

**Figure 2:**
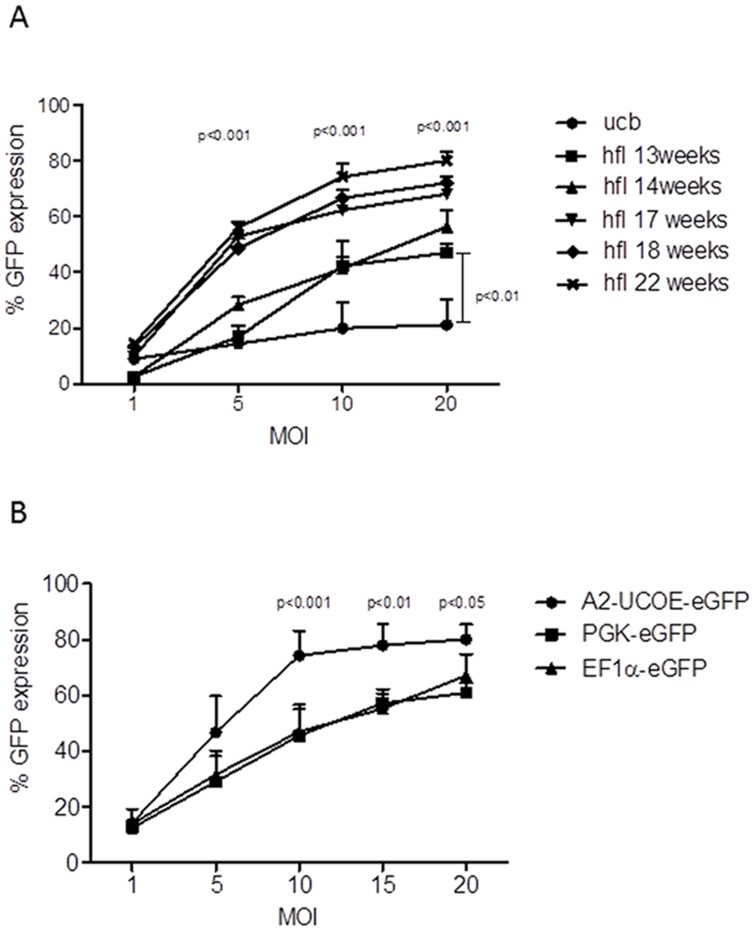
More efficient transduction of fetal liver-derived than cord blood-derived HSC. (A) Human fetal liver HSC are transduced more efficiently with the A2UCOE-eGFP LV than UCB-HSC, with higher efficiencies achieved with increasing gestational age from 13 through 22 weeks. (B) Higher levels of transduction of hflHSC were achieved with the A2UCOE-eGFP LV than with the PGK-eGFP and EF1α-eGFP vectors at differing multiplicities of infection (MOI).

LV transduction efficiency of hflHSC using the A2UCOE-eGFP construct (A2UCOE-LV) was 3.1 fold higher than UCB-HSC at MOI 20 for all gestational ages (n = 5 fetal samples ranging from 13 to 22 weeks gestation and 3 UCB samples; ANOVA, p<0.001). A 2.4 fold higher transduction efficiency in hflHSC (gestations 17-22 weeks) was observed over UCB-HSC at MOI 5 (p<0.001). We observed an increasing ability of the A2UCOE-LV to transduce hflHSC with increasing gestational age between 13 to 22 weeks. A 3.1 and 1.6 fold increase in levels of transduction efficiency in hflHSC from gestation 17-22 at MOI 5 and 20 respectively (p<0.001) compared to 13 weeks gestation was observed (Figure 2A).

Previous studies have shown that the A2UCOE can drive more reproducible and stable expression within adult bone marrow and peripheral blood cells compared to other frequently used elements such as the spleen focus forming virus (SFFV), cytomegalovirus (CMV) and EF1α promoters [20], [21]. However, in order to facilitate a more rational choice of promoter for targeting hflHSC, we decided to examine two other ubiquitous promoters that are frequently used in mammalian systems, namely EF1α and PGK and compare them to the A2UCOE.

Our results show that cells transduced with the A2UCOE-LV showed a significantly higher expression of the eGFP transgene in comparison to EF1α and PGK LVs, reaching a peak of 74.3±8.8% at MOI 10 (p<0.001), compared to 61±3.5% (p<0.05) and 67.2±7.5 respectively at MOI 20 (Figure 2B).

### Stable A2UCOE transgene expression in vitro

**Figure 3:**
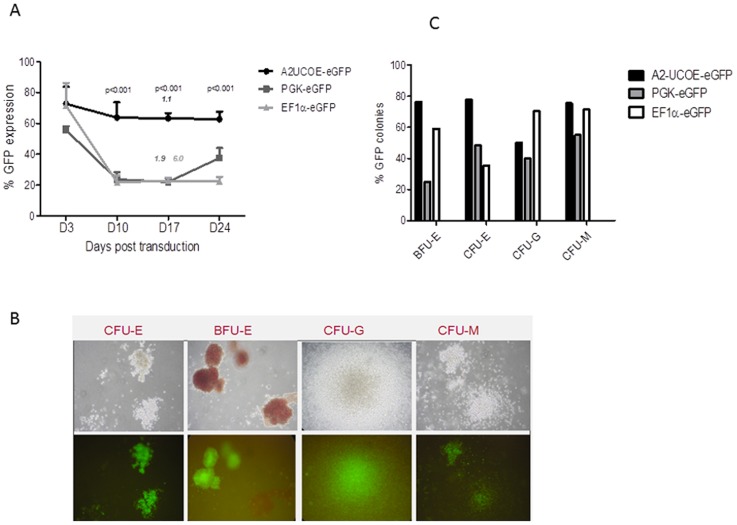
Sustained expression of A2UCOE-eGFP in *in vitro*. (A) hflHSC transduced with A2UCOE-eGFP, PGK-eGFP and EF1α-GFP LVs were maintained in culture for 24 days and analyzed at 3, 10, 17 and 24 days post-transduction. Expression from A2UCOE-eGFP was sustained over the 24 day period of culture. A rapid decline in PGK-eGFP (58% to 22%) and EF1α-eGFP (71% to 23%) expression was seen by Day 10. Average vector copy number per cell (VCN) at Day 17 of culture is given in italic text above the histogram lines. (B) A2UCOE-eGFP LV transduced cells showing maintenance of multilineage differentiation capability in methylcellulose culture assays at 17 days post-transduction. (C) Quantification of proportion of eGFP expressing colonies following differentiation of LV transduced hflHSC in semi-solid methylcellulose culture assays. Note comparable levels of eGFP-positive colonies with all three (A2UCOE, EF1α, PGK) LV types.

In order to determine the longevity of transgene expression with the different LV constructs, we transduced hflHSC with all three constructs to achieve initial levels of ∼60-70% eGFP positive cells with MOI of 10, 15 and 30 for A2UCOE-GFP, PGK-eGFP and EF1α-eGFP respectively. The percentage of GFP positive cells was largely stable throughout the 24 days of liquid culture for the A2UCOE-LV transduced cells, during which cell numbers expanded 2.5 fold. In contrast expression driven by the PGK and EF1α promoters resulted in a decrease in eGFP positive cells from 56±2.2 to 23±4.7% (p<0.001) and 71.5±14.6 to 21.7±4.4% (p<0.001) respectively by day 10 of culture and remained at this level untill the end of the period of culture at Day 24. The vector copy number per cell (VCN) at Day 17 post-transduction was 1.1±0.3, 1.9±0.2 and 6.0±0.5 for A2UCOE-GFP, PGK-eGFP and EF1α-eGFP respectively (Figure 3A).

In order to establish the efficacy of the A2UCOE in stabilizing transgene expression upon multilineage differentiation of hflHSCs, a colony forming assay (CFU) was performed. At Day 3 post-LV transduction, hflHSC were cultured in semi-solid methylcellulose media, and the number of total and eGFP-positive colonies was counted. CFU assays after semi-solid methycellulose culture on Day 3 LV transduced hflHSC showed the maintenance of multi-lineage differentiation capacity and expression of eGFP in all lineages (erythroid, granulocytes and myeloid) for all three vector groups, (Figure 3B-C). There was also a clear trend in the A2UCOE-eGFP LV transduced cells giving a higher percentage of eGFP-positive colonies compared to PGK-eGFP in the case of BFU-E and CFU-E and EF1α-eGFP in the CFU-E lineage (Figure 3C). In all other cases the percentage of eGFP-positive cells was comparable between the three different LVs.

### Stable A2UCOE-eGFP LV expression in hflHSCs *in vivo*

**Figure 4:**
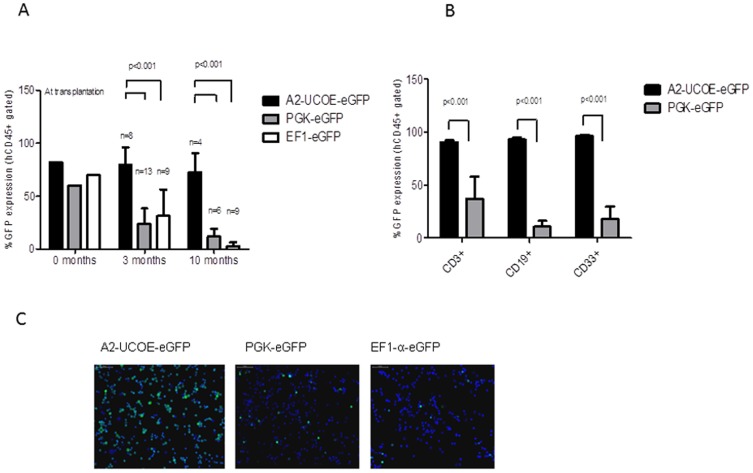
A2UCOE confers sustained transgene expression *in vivo*. (A) hflHSCs were transduced with either A2UCOE-eGFP, EF1α-eGFP or PGK-eGFP LVs and transplanted into sub-lethally irradiated NSG mice. A2UCOE-eGFP transduced hflHSC maintained transgene expression levels over 3 and 10 months post-transplantation whereas EF1α-eGFP and PGK-eGFP transduced cells lost eGFP expression rapidly. (B) Multilineage engraftment at 10-months post-transplantation from LV transduced hflHSC with higher percentage of eGFP-positive cells in A2UCOE-eGFP compared to EF1α-eGFP and PGK-eGFP. (C) Immunoflourescent staining of of bone marrow cells at 10 months post-transplantation showing sustained eGFP expression for A2UCOE-eGFP in comparison to PGK-eGFP and EF1α-eGFP.

We next conducted a long-term comparative analysis investigating stability of transgene expression *in vivo* by transplantation of LV-transduced hflHSC into irradiated newborn NOD-*SCID Il2rg^−/−^* (NSG) mice. hflHSC were transduced with A2UCOE-eGFP, PGK-eGFP and EF1α-eGFP LVs at different MOI to achieve a similar initial level of eGFP positive cells of ∼70% (Figure 2B). Following transplantation into newborn irradiated NSG mice, eGFP expression in peripheral blood cells was analyzed at 3 and 10 months post-reconstitution. Median levels of engraftment in peripheral blood were 3.6% (range = 0.2% to 25.2%), in 19 animals where data was available in the three groups of mice. Cells transduced with the A2UCOE-eGFP LV showed a sustained expression at 3 and 10 months post transplantation at 79.5±16.7% (n = 8) and 72.8±17.8% (n = 4) respectively. Contrastingly, PGK-eGFP and EF1α-eGFP transduced cells showed a rapid reduction in expression over 10 months of 5.1 (n = 6, p<0.001) and 22.2 fold (n = 9, p<0.001) respectively (Figure 4A). At the 10-month time point, peripheral blood samples from A2UCOE-eGFP transplanted mice showed a significantly higher and sustained eGFP expression in comparison to PGK-eGFP in all lineages including T (CD3+) (90.5±1.7% vs 37.2±20.6%; p<0.001), B (CD19+) (93±1.6% vs 10.5±5.7%; p<0.001) and myeloid (CD33+) (96.3±1.0 vs 17.8±11.9; p<0.001) cells (Figure 4B).

In order to assess the stability of expression in the hematopoietic stem and progenitor cells, mice were euthanized at 10 months post-engraftment and cells from bone marrow harvested and analyzed for eGFP expression. The percentage of eGFP positive cells was confirmed by fluorescence microscopy of bone marrow cells, reflecting the presence of a large population of eGFP expressing cells in the UCOE group, compared to fewer positive cells in the PGK as well as the EF1α groups (Figure 4C).

## Discussion

HSC-based gene therapy is gaining traction with a number of clinical trials targeting different conditions (X-SCID, ADA-SCID, ALD, CGD, Fanconi anemia, β-thalassemia) showing encouraging results [2], [3], [6], [27]-[30] and which have implications for curative lifelong treatment. Despite these clinical successes, problems of insertional mutagenesis and therapeutic gene silencing remain to be solved [16]. The discovery of the A2UCOE class of transcriptional regulatory element offers one option to overcoming these difficulties. The A2UCOE has been shown not to be susceptible to DNA methylation-mediated silencing and thus has the potential to bring about long-term transgene expression and therapy. Here we show the ability of an A2UCOE-based LV to efficiently transduce human fetal liver-derived HSC with maintenance of transgene expression over at least 10 months in an immunodeficient mouse model system, further underscoring its potential clinical utility.

The dominant chromatin opening and transcriptional activating capability of the A2UCOE has been shown to be able to drive reproducible and stable transgene expression in cell lines and more importantly mouse HSCs and their differentiated progeny *in vivo* [20], [21], [31]. In addition, the A2UCOE confers reproducible and stable transgene expression within human pluripotent cell populations and upon differentiation into cells representative of all three germ layers [22], [23]. Furthermore, this element is devoid of enhancer activity consisting of a methylation-free CpG island encompassing dual divergently transcribed promoters of housekeeping genes, which reduces the risk of insertional mutagenesis leading to host gene activation and oncogenesis [20]. The A2UCOE methylation-free CpG island is particularly potent at being able to resist silencing of transgenes [20], [32], [33] by negating promoter DNA methylation [21], [31].

The proliferative nature of fetal HSC makes these cells excellent targets for genetic modification using gene therapy vectors that require the proliferation of host cells. There is a high frequency of actively cycling HSC undergoing self renewal, which can be isolated from fetal livers with capacity to repopulate compared with HSC isolated from adult BM that by comparison are quiescent [34]-[36]. In line with findings from other groups [37]-[39], the mid-gestation fetal liver was found to contain an abundant source of CD34+CD133+ cells. These cells were found to be capable of generating various hemopoietic progeny in both *in vitro* and *in vivo* settings, confirming their HSC identity. Given that approximately 25,000 CD34+ HSC/kg bodyweight is required for hematopoietic reconstitution [40], a single harvest of fetal liver will yield adequate HSCs to treat an individual of up to 400 kg from the 10^7^ HSC derived.

Our data shows increased transduction efficiency of hflHSC increasing with gestational age over UCB-HSC at low MOI suggesting that hflHSC are highly amenable to LV-based genetic manipulation. Efficiency of lentiviral transduction in UCB-HSC was significantly lower under similar conditions. On the other hand, fetal stem cells have the ability to be transduced at a higher efficiency since they contain a higher proportion of actively cycling stem cells compared to cord blood or other adult sources [36], [41]. In addition, fetal HSC has also been shown to have a competitive engraftment advantage over adult HSC [42], [43]. Thus human fetal liver may be a potential source of abundant HSCs for prenatal and postnatal allogeneic transplantation as well as being a direct *in utero* gene therapy target.

While the A2UCOE promoter has been shown to be superior to the EF1α promoter when used to drive transgene expression in murine HSC, its performance has never been compared to other frequently used promoters such as the EF1α and PGK promoters in the context of clinically relevant primitive human HSCs. Hence, in this study we have evaluated the ability of three LVs driven by the A2UCOE, PGK and EF1α promoter elements to transduce and stably express from within hflHSC.

Reproducibility, levels and stability of expression is clearly controlled and dependant on the regulatory elements incorporated within the transgene cassette [16], [44]. Our data shows higher transduction efficiency and stable long-term transgene expression with the A2UCOE-eGFP LV across all MOI over that obtained with the PGK-eGFP and EF1α-eGFP vectors with hflHSC both *in vitro* and *in vivo* in mice, clearly suggestive of its utility for driving long-term expression.

hflHSC transduced with the A2UCOE-eGFP LV gave sustained eGFP expression within both bone marrow and all peripheral blood cell lineages. Maintenance of eGFP expression in T and B cells indicates the usefulness of the A2UCOE in targeting disorders of affecting these cell populations such as severe combined immunodeficiencies (SCID) [4] and Wiskott-Aldrich syndrome [45], [46]. Indeed, A2UCOE regulated LVs have been shown to fully rescue the disease phenotype of SCID-X1 [20] and CGD [31] at low (<1) vector copy number in mice. Likewise recombination activating gene 2 (*RAG2*) deficiency is an autosomal recessive disorder causing a complete lack of mature T and B lymphocytes leading to a SCID condition. Restoration of immune functions in Rag2 deficient mice by utilization of an A2UCOE-driven codon optimized human Rag2 sequence has been reported [47]. This is suggestive of an optional vector choice for clinical implementation.

In conclusion, our data has shown that hflHSC represent a rich source of HSC. They are highly amenable to genetic manipulation with LVs and transduce more efficiently than UCB-HSC. We have also shown sustained eGFP reporter gene expression and ability to maintain expression in multiple hematopoietic lineages in mice engrafted with A2UCOE-eGFP LV transduced cells. Owing to the silencing resistant property of A2UCOE-regulated transcription units, it represents a superior choice over the PGK and EF1α promoters for gene therapy targeting HSC.
